# Revealing antibiotic resistance’s ancient roots: insights from pristine ecosystems

**DOI:** 10.3389/fmicb.2024.1445155

**Published:** 2024-10-09

**Authors:** Rubén Agudo, M. Paloma Reche

**Affiliations:** Facultad de Farmacia, Universidad San Pablo-CEU, CEU Universities, Madrid, Spain

**Keywords:** pre-antibiotic era, antibiotics, resistome, antibiotic resistance genes, horizontal gene transfer, pristine environments

## Abstract

The prevailing belief that antibiotic resistance mechanisms emerged with human antibiotic use has been challenged. Evidence indicates that some antibiotic resistance genes (ARGs) have a long evolutionary history, predating the advent of antibiotics in human medicine, thereby demonstrating that resistance is an ancient phenomenon. Despite extensive surveys of resistance elements in environments impacted by human activity, limited data are available from remote and pristine habitats. This minireview aims to compile the most relevant research on the origins and evolution of ARGs in these habitats, which function as reservoirs for ancient resistance mechanisms. These studies indicate that ancient ARGs functionally similar to modern resistance genes, highlighting the general role of natural antimicrobial substances in fostering the evolution and exchange of diverse resistance mechanisms through horizontal gene transfer over time. This minireview underscores that antibiotic resistance was present in ancestral microbial communities and emphasizes the ecological role of antibiotics and resistance determinants. Understanding ancient ARGs is crucial for predicting and managing the evolution of antibiotic resistance. Thus, these insights provide a foundational basis for developing new antibiotics and strategies for microbial resistance management.

## Graphical abstract

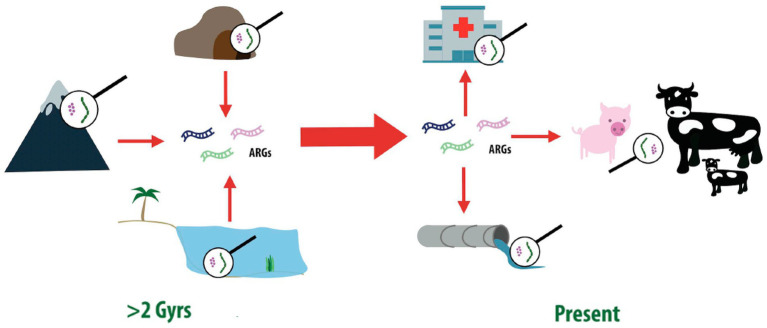

## Introduction

Antibiotics are considered one of the most remarkable discoveries in the history of medicine. The mass production and introduction of antibiotics in clinical practice during the 1940s represent one of the most important interventions in the control of infectious diseases enhancing the quality of life and life expectancy.

Despite their groundbreaking impact, the widespread use of antibiotics has led to an unintended consequence: the growing development of bacterial resistance to antibiotics, which has become a global threat and an issue of great concern in the clinical practice. However, evidences demonstrate that bacteria already possessed resistance mechanisms prior the discovery and introduction of antibiotics into clinical practice (Perry et al., 2016) indicating that it is not a recent phenomenon. Thus, antibiotic resistance is inherent, ancestral and deeply rooted in the microbial pangenome (D'Costa et al., 2011).

This review focuses on the results of the research of these extreme natural habitats, including deep subsurface terrestrial depths, deep oceanic depths (Brown and Balkwill, 2009) as well as in ancestral settings such as isolated caves (Bhullar et al., 2012; Pawlowski et al., 2016) or permafrost (Afouda et al., 2020; Chen et al., 2016; D'Costa et al., 2011; Segawa et al., 2013). Exploring the functionality of antibiotic resistance genes (ARGs) discovered in ancient settings provides a distinctive insight into the origins and evolution of antibiotic resistance in bacteria (Hibbing et al., 2010; Lupo et al., 2012; Sultan et al., 2018). This exploration holds a dual significance: Firstly, it documents that the extensive environmental collection of resistance genes show the ability to produce and tolerate antibiotics, even before their clinical use, through mechanisms that are similar or identical to those described in pathogenic bacteria today. Secondly, understanding this evolutionary process is crucial for predicting, preventing and managing the significance of currently emerging or evolving resistance genes (Perry et al., 2016; van der Kolk, 2015).

## Exploring the resistome: origins and evolution of antibiotic resistance genes

Numerous studies have explored and described the prevalence of antibiotic resistance genes in different environments revealing an ubiquitous and widespread nature (Bengtsson-Palme et al., 2018; von wintersdorff et al., 2016; Xiao et al., 2023). The collection of genes involved in antibiotic resistance across both pathogenic and non-pathogenic bacteria is called “resistome.” This term includes those genes with the potential to become ARGs under the appropriate selection pressure, being a primary external source of new antibiotic resistance patterns (Wright, 2007). It is now widely assumed that the environmental resistome is the origin and reservoir of ARGs (D'Costa et al., 2007; Finley et al., 2013; Forsberg et al., 2012; Kim and Cha, 2021). Soil, proposed as the most important natural reservoir of ARGs on Earth (Nesme et al., 2014), has been studied aiming to understand the environmental factors controlling ARGs abundance and diversity from local to global scales (Delgado-Baquerizo et al., 2022). These genes likely evolved in environments populated by a myriad of different microorganisms engaged in constant relationships and competitions, influenced by the presence of natural antibiotics, which are believed to have evolved over millions of years proving that antibiotic resistance is an ancient phenomenon (Baltz, 2008; Barlow and Hall, 2002; Hall and Barlow, 2004).

ARGs provide a selective advantage as weapons of microbial warfare against resource competitors. Moreover, antibiotics are recognized as signaling molecules in microbial ecosystems at subinhibitory levels (Davies et al., 2006; Linares et al., 2006) impacting dynamics in natural microbial communities. Antibiotic compounds can function as transcriptional regulators, promoting quorum sensing and biofilm formation processes (Hibbing et al., 2010; Newton et al., 2023). Additionally, they can exert a role in virulence factor production and influence host-pathogen interactions (Sengupta et al., 2013). Therefore antibiotic resistance can have arisen through gene duplication and genetic diversification via mutations leading to the emergence of proteins with distinct physiological functions under selective pressure (Sandegren and Andersson, 2009). Therefore, precursor proteins possesses some affinity for specific antibiotics, gradually evolving into robust antibiotic-protein interaction and ultimately giving rise to resistance mechanisms, resulting in the efficient resistance genes observed in the resistome today (Dantas and Sommer, 2012).

An example of this mechanism of exaptation involves the acquisition of serine *β*-lactamases from D-peptidases involved in bacterial cell wall biosynthesis (Meroueh et al., 2003). Another example is the plasmid-mediated quinolone resistance exerted by *qnr* gene family: Fluoroquinolones bind to the DNA gyrase-DNA complex, stabilizing the cleaved complex and ultimately leading to lethal double-stranded breaks (Drlica et al., 2008). However, Qnr proteins have been found to bind *E. coli* DNA gyrase protecting it from quinolone inhibition (Jacoby et al., 2014). It is postulated that the original physiological role of *qnr* genes may have been to shield DNA gyrase from toxic substances and modulate gene expression in response to environmental changes (Hernandez et al., 2011). Moreover, efflux pump genes are also frequently present in antibiotic-producing bacteria (Martinez, 2009; Nikaido and Pages, 2012). In addition to their physiological role associated with cell homeostasis, these genes are frequently located within biosynthetic gene clusters responsible for antibiotic production, thus enabling the producing organism to expel antibiotics and consequently imparting resistance to the producing organism. Additionally, mutations in the promoter or regulatory expression sequences of certain efflux pump genes, along with their duplication, might lead to their overexpression and promote drug resistance (Sandegren and Andersson, 2009).

## Antibiotic resistance in the pre-antibiotic era

Environments characterized by minimal anthropogenic contamination, unaffected by the selective pressure of antibiotics introduced into the clinical practice during the 20th century, offer insights into the spectrum of resistance mechanisms prior to the influence of these factors (Martinez, 2009). These studies underscore the broad and ancient presence of antibiotic resistance, highlighting how resistance mechanisms have been an integral part of microbial ecosystems long before the advent of modern antibiotics. Most of the Phylogenetic and experimental studies of ARGs have been performed on *β*-lactamases. The evolutionary trajectories of serine-β-lactamases and metallo-β-lactamases indicate their ancient origins dating back over 2 billion years (Fevre et al., 2005; Garau et al., 2005; Hall and Barlow, 2004; Song et al., 2005). This hypothesis has been reinforced by obtaining precambrian *β*-lactamases genes (2–3 billion years old) by ancestral protein reconstruction that displayed a promiscuous pattern of antibiotic degradation, including third-generation cephalosporins (Risso et al., 2013).

The detection and characterization of antibiotic-resistant bacteria in pristine environments has been predominantly documented in untouched Arctic and Antarctic cryosols, as well as in permafrost (Afouda et al., 2020; Allen et al., 2009; D'Costa et al., 2011; Haan and Drown, 2021; Kashuba et al., 2017; Mindlin et al., 2008; Perron et al., 2015; Petrova et al., 2014; Rigou et al., 2022; Van Goethem et al., 2018).

Specifically, D’Costa et al. conducted metagenomic analyses on ancient DNA extracted from late Pleistocene (30,000-year-old) Beringian permafrost sediments. They identified a repertoire of genes conferring resistance to *β*-lactam, tetracycline, and glycopeptide antibiotics, including vancomycin. Structural and functional studies on the complete vancomycin resistance element VanA confirmed its similarity to modern variants (D'Costa et al., 2011).

Perron et al. (2015) demonstrated the presence of diverse functional mechanisms of antibiotic resistance in bacteria dating back at least 5,000 years. Through a functional metagenomic screen of bacteria isolated from permafrost, they identified eight resistance genes targeting clinical aminoglycoside, *β*-lactam, and tetracycline antibiotics. These included three efflux pump-related genes, two inactivating enzyme genes, and three involved in membrane modification or synthesis. Among the five genes conferring resistance to aminoglycosides, four provided resistance or cross-resistance against amikacin, the first semisynthetic aminoglycoside developed to counteract resistance to natural aminoglycosides such as streptomycin and siomicin (Kawaguchi, 1976). Several of the resistance genes identified in this study exhibited striking similarity to those found in contemporary pathogenic bacteria. Furthermore, functional resistance genes were detected in bacterial genera known for antimicrobial production, as well as in bacteria typically not associated with antimicrobial production.

In another study, Kashuba et al. analyzed permafrost soil from Yakutia, Russia dating back 3.5 million years. The study describes the isolation of two *Staphylococccus* strains that were identified as *Staphylococcus warneri* and *Staphylococcus hominis*. The *S. hominis* isolate possessed multiple genes conferring resistance to the aminoglycoside, β-lactam, MLS (macrolide, lincosamide and streptogramin B) and phenicol groups of antibiotics (Kashuba et al., 2017).

Mindlin et al., documented ancient bacteria resistant to various antibiotics found in Arctic permafrost subsoil sediments in Siberia of several genesis and age. They identified resistance genes in bacteria dating back up to 2 to 3 million years, including strA–strB genes encoding aminoglycoside phosphotransferases, commonly found in contemporary bacterial strains within transposons and plasmids, and aadA genes encoding adenylyltransferases, typically encountered as gene cassettes in composite transposons (Mindlin et al., 2008).

The study conducted by Van Goethem et al., employed a metagenomic approach in 17 “antibiotic naïve” Antarctic soils revealing 177 ancestral genes, mainly in the *Bacteroidetes and Acidobacteria* phyla that potentially could confer resistance to natural antibiotics. They concluded that most of the genes described would have their origin in antibiotic-producing species, a theory supported by the presence of antibiotic biosynthesis genes in the phyla harboring resistance genes and the absence of mobile genetic elements. The most abundant ARGs identified were undecaprenyl pyrophosphate phosphatases, conferring bacitracin resistance, and genes encoding efflux pumps, particularly macrolide transporters ATP-binding/permease proteins (Van Goethem et al., 2018).

In addition to the findings in permafrost and arctic soils, Pawlowski et al. perform different assays to describe the intrinsic resistome of *Paenibacillus* sp. LC231 strain in Lechuguilla Cave (New Mexico, United States). This bacterial isolate presented resistance to most clinically used antibiotics (Pawlowski et al., 2016). The study revealed 18 chromosomal resistance elements, including five novel determinants and three previously unknown resistance mechanisms. Comparison with related surface *Paenibacillus* sp. confirmed resistance conservation over millions of years, indicating the longevity of these genes within the genus. A previous study by the same group describes the ancient Lechuguilla Cave’s microbiome, revealing bacteria resistant to 14 antibiotics, including daptomycin. Enzyme-mediated resistance mechanisms are described against macrolide antibiotics, indicating the prevalence of resistance even in environments untouched by human antibiotic use (Bhullar et al., 2012).

Thus, microbial communities inhabiting remote and pristine soils shielded from the physical and biological disturbances experienced at the surface for millennia, provide valuable genetic reservoirs for exploring the evolutionary origins of natural antibiotic resistance since the pre-antibiotic era (Allen et al., 2010; Segawa et al., 2013), providing information on the potential composition of the resistome in its original state (Steven et al., 2006).

On the other hands, Larsen et al., showed that particular lineages of methicillin-resistant *Staphylococcus aureus* appeared in European hedgehogs before the human use of antibiotics. They also revealed that the hedgehog dermatophyte *Trichophyton erinacei* produces two *β*-lactam antibiotics that provide a natural selective environment in which methicillin-resistant *S. aureus* isolates have an advantage over susceptible isolates (Larsen et al., 2022). These results suggest that methicillin resistance emerged before human use of β-lactams utilization as a co-evolutionary adaptation of *S. aureus* to the colonization of dermatophyte-infected hedgehogs.

These studies definitively demonstrate that the prevalence of antibiotic resistance is not directly correlated with human antibiotic usage.

## Discussion

The acquisition and dissemination of ARGs among pathogenic bacteria poses a serious challenge to the effective treatment of bacterial infections in humans and domestic animals (Karkman et al., 2019; Zhu et al., 2019). This tendency overcomes the rate of new drug development and undermines the progress made by the use of antibiotic treatment in modern medicine.

The resistome concept has been widely used and studied in different environments revealing that ARGs are ubiquitous and widespread (Li et al., 2015; Nesme et al., 2014). Soil resistome are clinically significant as they facilitate bidirectional transfer of resistance genes between soil and pathogenic bacteria (Forsberg et al., 2012). Understanding the prevalence and distribution of environmental ARGs is of particular interest as they represent a source of emerging antibiotic resistance mechanisms that could be acquired by pathogenic bacteria (Afouda et al., 2020; Martinez et al., 2015; von Wintersdorff et al., 2016). Likewise, the diversity of the soil resistome can explain the rapid evolution of resistance against modern antibiotics, including the semisynthetic ones (Perron et al., 2015). Transfer of antibiotic resistance genes can occur through mobile elements, facilitating horizontal gene transfer among bacteria occupying the same ecological niches and promoting the spread of resistance across bacteria species across clinical, domestic, or wildlife environments (Lee et al., 2020).

While there is an evident positive correlation between the prevalence of clinically significant resistance genes and the proximity to anthropogenic activity (Nardelli et al., 2012; Thaller et al., 2010), experimental evidence also indicates that certain ARGs originated millions of years ago, predating the discovery and implementation of antibiotics in mid-20th century (Bhullar et al., 2012; D'Costa et al., 2011; Perron et al., 2015; Perry et al., 2016). Moreover the present understanding of ancient antibiotic resistance reveals dynamic genetic pools capable of transmission across bacterial species (Dantas and Sommer, 2012).

Therefore, examining ancient microbes from environments, unaffected by human activity and antibiotic use, provides valuable insights into the evolutionary trajectory of ARGs and the origins and evolution of resistance mechanisms. The results of the study by D’Costa et al. carry important implications for understanding antibiotic resistance evolutionary and complex phenomenon. It underscores that antibiotic resistance is an ancestral event that exist way before the human use of antibiotics. Moreover, the advent of clinical antibiotics has provided a selective environment that has fueled the dissemination and diversification of ARGs.

In this regard, natural environmental conditions differ significantly from those created by human activities such as clinical medicine, agriculture, and animal husbandry, where antibiotics are produced in concentrations orders of magnitude higher, for longer durations, and on a much higher scale (Spagnolo et al., 2021). Since 1940, antibiotics have become ubiquitous pollutant in all types of environments (Kraemer et al., 2019). Recent studies indicate that even low levels of antibiotic pollution can select for high levels of resistance (Wistrand-Yuen et al., 2018). Consequently, the industrial-scale use of antibiotics worldwide has led to a continuous influx of these compounds into the environment, preventing natural systems from returning to baseline levels. This intense and persistent selective pressure is absent in natural untouched environments, which explains why resistance traits have not become common or deeply ingrained in bacterial populations over millions of years.

In his context, resistance genes found in pristine environments exhibit striking similarities to those found in modern bacteria, evidencing the conservation and transmission of resistance mechanisms throughout evolution and providing a selective advantage (Afouda et al., 2020; D'Costa et al., 2011). The presence of multidrug-resistant organisms in unspoiled environment reinforces the notion that ARGs are ancestral and widespread, indicating that competition for resources likely plays a dominant role in species survival. This competition occurs through different adaptations, including the production of antimicrobials and resistance mechanisms to outcompete nutritional rivals (Bhullar et al., 2012).

The production of antibiotics and resistance determinants is ubiquitous in bacteria in which resistance gene products neutralize or detoxify the secondary metabolites produced by the same or neighboring cells. Resistance genes are often linked to and co-regulated with the antimicrobial biosynthesis genes. Hence, the widespread occurrence of antibiotic resistance in environmental bacteria is an important indicator of antibiotic production, as seen in soil ecosystems (D'Costa et al., 2006; Nodwell, 2007). Antibiotics likely serve an ecological role to prevent the growth of other competitors in the same ecological niche. As a result, antibiotic-producing microorganisms and coexisting species must develop protection mechanisms, leading to the appearance of resistance. These resistance determinants, or proto-determinants, were genes with diverse physiological functions that evolved to acquire antibiotic resistance as a new function (Galan et al., 2013; Perry et al., 2014).

Indeed, it is now postulated that these genes would have spread from environmental microorganisms to human commensal organisms and from them, to pathogens (Wright, 2010). This process of transfer from the environment to the human environment is stochastic and, therefore, the more prevalent a resistance mechanism is in the environment, the more likely it is to be transferred. The existence of reservoirs of resistance in the environment can significantly accelerate bacterial evolution towards multidrug resistance (Forsberg et al., 2012) so it is crucial to consider the vast diversity of ARGs within microbial populations when developing or implementing new antibiotic strategies (Perron et al., 2015).

Further in-depth investigations into the environmental resistome are crucial. These investigations not only aid in revealing precursors and gaps in the evolutionary history of antibiotic resistance genes but also possesses predictive value, serving as a critical tool for identifying potential threats posed by resistance to new antibiotics introduced in the clinical practice, serving as an early detection system (von Wintersdorff et al., 2016). Given the broad and ancient nature of the resistome, the existence of highly evolved and efficient antibiotic resistance mechanisms could be anticipated. However, although many putative resistance genes have been identified, it remains difficult to predict which of these mechanisms will successfully transfer to and be expressed in pathogenic bacteria (Christaki et al., 2020). Therefore, acknowledging the ancient origins of the resistome and understanding it, would significantly enhance our comprehension of the evolutionary pathways of antibiotic resistance and the development of new antibiotic drugs. Moreover, because the transfer and expression of resistance mechanisms in pathogens remain difficult to anticipate, further research is urgently needed to better assess potential risks, improve predictive models, and develop more effective strategies for combating the increasingly pressing challenge of antibiotic resistance.
